# Anthocyanin-loaded complexes of glycated dual milk-derived proteins: thermal stability, storage stability, and simulated digestion

**DOI:** 10.1016/j.ultsonch.2025.107485

**Published:** 2025-07-28

**Authors:** Shuangshuang Wang, Zhenzhu Li, Qingshuang Qiu, Huilin Lv, Haokun Zhang, Hang Wang, Rongzu Nie, Wupeng Ge, Peifeng Li

**Affiliations:** a College of Food and Bioengineering, Zhengzhou University of Light Industry, Zhengzhou 450001, China; b Key Laboratory of Cold Chain Food Processing and Safety Control, Ministry of Education, Zhengzhou University of Light Industry, Zhengzhou 450001, China; c Institute of Life and Health, Zhengzhou University of Light Industry, Zhengzhou 450001, China; d College of Food Science and Engineering, Shaanxi Engineering Research Centre of Dairy Products Quality, Safety and Health, Northwest A&F University, Yangling 712100, China

**Keywords:** Anthocyanins, Ultrasonic treatment, Nanocomplexes, Stability, Encapsulation

## Abstract

Anthocyanin (ACN) have attracted considerable scholarly attention owing to exceptional biological activities. Nonetheless, their limited stability and bioavailability present significant challenges to practical applications. To enhance the thermal stability, storage stability, and simulated digestion of ACN, we utilized whey protein isolate nanofibrils-casein (CA) complexes (WPCA) and WPCA– fucoidan (FD) glycated conjugates (WPCA-FD), as developed in our previous study, to formulate ACN-loaded complexes. These complexes, referred to as Complex I (ACN@CA), Complex II (ACN@WPCA), and Complex III (ACN@WPCA-FD), were synthesized using ultrasonic treatment. The average particle sizes of these complexes were determined to be 102.1 nm, 175.5 nm, and 365.3 nm, respectively. The ACN encapsulation efficiency of Complex III (59.47 %) increased almost 18 % than Complex I, while Complex III had the highest zeta potential (−35.25 mV). Scanning electron microscopy (SEM) analysis revealed that the surface of Complex III displayed an increased overall size and distinct morphological features, characterized by a uniform, regularly spherical shape with a relatively smooth texture. Fourier-transform infrared (FTIR) spectroscopy verified that the formation of complexes was facilitated by hydrogen bonding and hydrophobic interactions, with a notable hydrogen bond interaction occurring between ACN and WPCA-FD. Furthermore, the protective effect (thermal and storage stability) of Complex III was more significant, showed slower ACN release in simulated releasing environment *in vitro.* This study demonstrated significant potential for enhancing the applicability of stabilizing anthocyanin in developmental processes.

## Graphical abstract

**Figure:**
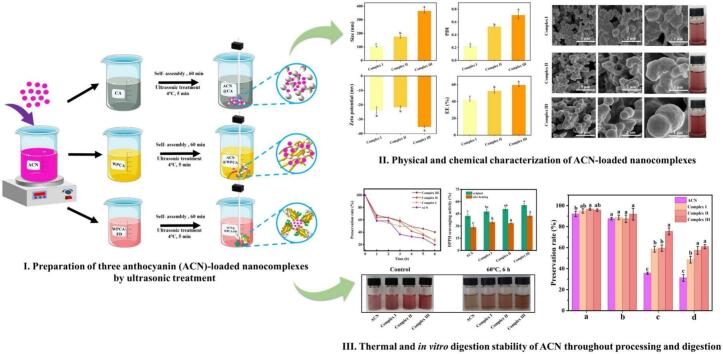

## 1 Introduction

Anthocyanin (ACN) is a flavonoid polyphenolic compound characterized by multiple phenolic hydroxyl groups, predominantly found in various pigmented foods, and is renowned for its potent antioxidant, anti-inflammatory, anti-obesity, and other advantageous biological activities [[1], [2], [3]]. Nevertheless, the stability of ACN is susceptible to external environmental factors and numerous physiological barriers, including structural composition, processing methods, metal ions, pH, temperature, enzymatic activity, oxygen, and light [4,5]. Temperature, in particular, exerts a substantial influence on the stability of anthocyanin, with increased temperatures leading to a reduction in stability [6]. Conventional heat treatments can alter the color, chemical stability, and nutritional properties of ACN. Furthermore, as a hydrophilic compound sensitive to pH, digestive enzymes, and other gastrointestinal conditions, ACN are chemically prone to nucleophilic attack [7]. Research has demonstrated that the type, quantity, and position of substituents on the anthocyanin 2-phenylbenzopyrane cationic parent nucleus significantly influence its stability [8]. Furthermore, hydroxylation processes facilitate the *in vivo* degradation of the anthocyanin prototype, leading to the generation of low-molecular-weight degradation products, such as phenolic acids, which adversely affect its stability [9]. This degradation consequently reduces the compound's efficacy and constrains the full manifestation of its biological effects.

To overcome these challenges, various encapsulation strategies have been utilized. Notably, protein-polysaccharide complexes synthesized from glycosylated modified proteins in combination with carboxymethyl cellulose have been reported to effectively mitigate anthocyanin color degradation and improve both the retention and thermal stability of ACN during thermal processing [10]. Studies have demonstrated that protein-polysaccharide covalent complexes synthesized via the Maillard reaction are effective in mitigating protein aggregation and precipitation under conditions of high ionic strength or low pH [11]. Moreover, these complexes contribute to increased protein stability within the gastrointestinal digestive environment and enhance the encapsulation and protection of ACNs, thus preventing their premature release and degradation in the gastrointestinal tract [12]. Previous studies conducted by our research team have also shown that whey protein isolate nanofibrils-casein (CA) complexes, which are dual-natural milk-derived proteins (WPCA), can be conjugated with fucoidan (FD) using dielectric barrier discharge cold plasma treatment [13]. The resulting WPCA-FD glycosylation products exhibit dual protein networks that function as reservoirs for bioactive compounds, thereby positioning them as promising candidates for carrier materials. Therefore, it is expected that these conjugates of cold plasma modification protein-polysaccharide will function as potential nanocarriers, efficiently encapsulating and protecting hydrophilic bioactive compounds [14,15]. Research on the development of delivery systems and the enhancement of the stability of encapsulated ACN has emerged as a prominent area of interest. However, further investigation is required into the use of protein-polysaccharide complexes as carrier materials for the encapsulation and protection of anthocyanin.

Recently, ultrasound-assisted methodologies have emerged as a potent approach to augment the binding interactions between ACNs and encapsulating materials, thereby facilitating the formation of stable nanocomplexes with reproducible structures [[16], [17], [18]]. In this investigation, ultrasonic treatment was systematically employed as a standardized technique to ensure uniform nanocomplex assembly, thus enabling a direct comparison of the functional performance conferred by various carrier matrices (CA, WPCA, and WPCA-FD). We postulated that the ultrasonically treated WPCA-FD conjugate would surpass CA and WPCA in enhancing the thermal stability, storage stability, and resistance to simulated digestion of ACNs. This systematic approach also permits a critical evaluation of the role of glycosylation (via FD conjugation) in improving stability under standardized ultrasonic conditions. The study examined the effects of three complexes (CA, WPCA, and WPCA-FD) on the thermal stability, storage stability, and bio-accessibility of ACN during processing and digestion. A particular focus is placed on employing ultrasonic treatment uniformly as a standardized method to ensure reproducible complexes assembly, enabling direct comparison of the functional performance imparted by different carrier matrices.

## 2 Materials and methods

### 2.1 Materials

Mulberry anthocyanin (ACN) with a purity of 25 % was sourced from Tianjin Jianfeng Natural Product R&D Co., Ltd. in Tianjin, China. Casein, with a purity of at least 80 %, was procured from Sigma-Aldrich in Shanghai, China. Fucoidan (FD), exhibiting a purity of 98.3 % and comprising 25.8 % L-Fucose and 26.8 % sulfated content, was acquired from Shandong Jiejing Co., Ltd. in Rizhao, China, and is derived from *Fucus vesiculosus*. Pancreatin, pepsin, Bile salts and α-amylase were obtained from Solarbio Science & Technology Co., Ltd. (Beijing, China). All other chemicals utilized in this study were of analytical grade and were obtained from Sigma-Aldrich, unless stated otherwise.

### 2.2 Preparation of three different anthocyanin-loaded complexes

**Scheme 1:**
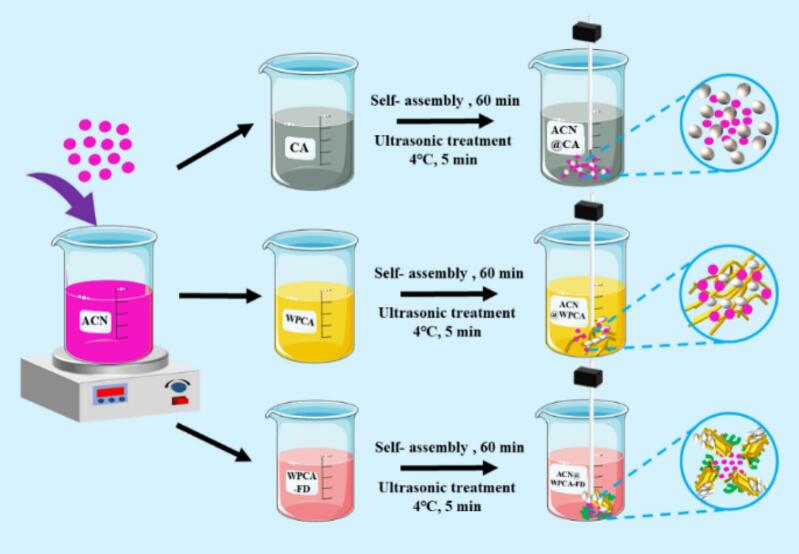
Schematic diagram of preparation of anthocyanin nanocomposites.

CAs powders were suspended in deionized water (DW) at a concentration of 1 % total solids (w/v). The suspension was maintained at a pH of 11.5 using 1.0 M NaOH and subsequently stirred at 500 rpm for 2 h to ensure complete dissolution. Subsequently, the CAs solution was neutralized to a pH of 5.0, after which ACN were added to form the Complex I (ACN@CA). The preparation of WPCA and WPCA-FD conjugate is based on the method described in our previous work [19]. Based on the preparation of WPCA-FD conjugate, the construction of other nanocomplexes formed were Complex II (ACN@WPCA) and Complex III (ACN@WPCA-FD). Briefly, ACN was dissolved in deionized water and passed through a 0.22 μm filter membrane to remove the larger insoluble impurities. Then, CA, WPCA, and WPCA-FD was added into the solution (sample: ACN ratio was 6:1), using the methods described in our previous work [15]. The pH of the mixture was adjusted to 3.5 using hydrochloric acid (0.1 M), and the mixture was stirred at room temperature for 60 min. Each sample was subjected to a uniform shearing process (10,000 rpm, 5 min). The mixture was then homogenized using a 13 mm ultrasonic probe (Scientz-IIZ, Ningbo, China) at 540 W in an ice bath for 5 min (2 s turning on/2 s turning off) to obtain the ACN-loaded complexes (Scheme 1). The ACN solution was mix with deionized wat in that above ratio to obtain a solution of the same concentration as a control.

### 2.3 Total anthocyanin

Total anthocyanin were measured according to previously reported pH differential method [20]. The samples were diluted to appropriate multiples in pH 1.0 buffer (0.025 M) and pH 4.5 buffer (0.4 M), and absorbance was measured at wavelengths of 520 nm and 700 nm.

The content (C) of total anthocyanin (mg/L) was calculated using equation (1–2):

(1) $$\text{A=Ap}{\text{H}}_{\text{1.0}}\text{(}{\text{A}}_{\text{520nm}}\text{-}{\text{A}}_{\text{700nm}}\text{)-Ap}{\text{H}}_{\text{4.5}}\text{(}{\text{A}}_{\text{520nm}}\text{-}{\text{A}}_{\text{700nm}}\text{)}\;$$

(2) $$\text{C (mg/L)=}\frac{\text{A}\times \text{DF}\times \text{Mw}}{\varepsilon \times \text{L}}$$

Where *ApH1.0* is the λ max of the sample diluted with pH 1.0 buffer.

*ApH4.5* is the λ max of the sample diluted with pH 4.5 buffer.

*Mw* was the molar molecular mass of procyanidin −3-O- glucoside (C3G) (449.2 g/mol).

*DF* is the dilution multiple of the sample.

*ε* is the molar extinction coefficient of cyanidin −3-O- glucoside (C3G) (26 900 L/mol cm^−1^).

*L* is the optical path length (1 cm).

### 2.4 Encapsulation efficiency

Encapsulation efficiency (EE) was determined by ultrafiltration centrifugation, as described in our previous work [15]. Prior to conducting the analysis, samples were standardized to an equivalent total anthocyanin content, as quantified by the pH-differential method detailed in Section 2.3, to ensure uniform baseline conditions. The samples were placed in ultrafiltration tubes with a molecular weight cutoff of 3 kDa, which facilitated the complete retention of encapsulated ACNs while permitting the passage of free anthocyanins. Centrifugation was then executed at 5000 *g* for 30 min at 4 °C, a condition previously optimized to prevent the disruption of nanoparticles. The concentration of free ACNs in the filtrate was determined according to the procedure described in Section 2.3, and EE was subsequently calculated using equation (3).

(3) $$\text{EE (\%)}=\frac{\text{Total ACNs-Free ACNs}}{\text{Total ACNs}}\times \text{100}\;$$

Where Total ACNs represented the amount of anthocyanin added to the samples; Free ACNs represented that the amount of anthocyanin in the filtrate.

### 2.5 Dynamic light scattering (DLS)

The particle size, polydispersity index (PDI), and zeta potential of the ACN-loaded complexes were characterized by dynamic light scattering (DLS) following a previously reported method [21]. The distilled water (DW) was used as a dispersant (refractive index = 1.330, viscosity = 1.002 mPa s). The solutions of samples were diluted by DW to an identical concentration of 0.1 % (w/v). Measurements were conducted at room temperature (25 °C) with a detection angle of 173° using a Zetasizer Nano-ZEN3600 analyzer (Malvern Instruments Ltd., UK).

### 2.6 Field emission-scanning electron microscopy (FESEM)

The microstructure of the anthocyanin-loaded complexes was evaluated using a SU8600 cryo-scanning electron microscopy (Cryo-SEM, Hitachi, Tokyo, Japan), operated in the secondary electron mode with an accelerating voltage of 5 kV, according to the method described by Tie, et al. [22] with some modifications.

### 2.7 Fourier transform infrared spectroscopy (FTIR)

The FTIR analysis was carried out according to our previous study [23]. Freeze-dried powder sample (1 mg) and KBr (100 mg) were mixed, pressed, and analyzed using an FTIR spectrometer (Vertex 70, Bruker Ltd., Ettlingen, Germany). To mitigate the effects of noise interference and mechanical drift, the acquired spectra underwent autonomic baseline correction, smoothing, and normalization processing. The following spectrometer parameters were as follows: the scan range of 4000–400 cm^−1^ with 64 scans and a resolution of 4 cm^−1^ for each sample.

### 2.8 Measurement of thermal stability

The thermal stability of the complexes was evaluated using a well-established method [16]. The thermal stability of ACN was assessed at a temperature of 60 °C. Samples were placed in glass vials equipped with screw caps and subjected to heat treatment in a water bath maintained at 60 °C for a duration of 6 h. The retention of ACN in the samples was evaluated through hourly sampling. Free ACN served as the blank control. The content of ACN was determined using the pH differential method, as detailed in Section 2.3. The retention rate was calculated in accordance with equation (4).

(4) $$\text{Retention rate}\;\left(\text{\%}\right)=\frac{{\text{C}}_{\;\text{t}}}{{\text{C}}_{\text{0}}}\times 100$$

Where C_0_ and C_t_ represent the ACN content at initial and time t, respectively.

### 2.9 Assessment of storage stability of anthocyanin complexes

The assessment of storage stability was conducted following the methodology outlined by Jiang, et al. [24], with necessary modifications. The samples were placed in glass bottles equipped with screw caps, sealed, and stored at ambient conditions under normal lighting for a duration of 70 days. Sampling was performed at 10-day intervals to evaluate the retention rate of ACN within the samples. The retention rate was measured using the procedure detailed in Section 2.8, employing free anthocyanin as the blank control.

### 2.10 Evaluation of the antioxidant capacity of anthocyanin complexes

The DPPH radical scavenging activity of the anthocyanin nanocomplexes was determined after continuous heat treatment based on the method described in our previous work [15]. In summary, 2 mL aliquots of the sample solution (5 mg/mL) or deionized water were combined with equal volumes of a DPPH solution and incubated in darkness for 30 min. Following incubation, the absorbance of the reaction mixture was measured at 517 nm. The free radical scavenging activity was quantified using equation (5).

(5) $$DPPH\;scavenging\;activity\left(\text{\%}\right)=\frac{{\text{A}}_{\text{c}}\text{-}{\text{A}}_{\text{s}}}{{\text{A}}_{\text{c}}}\times \text{100}$$

### 2.11 Extracorporeal gastrointestinal digestion of anthocyanin complexes

Referring to previous studies, *in vitro* gastrointestinal digestion experiments were conducted utilizing a three-step simulated digestion model, which encompasses the oral cavity, stomach, and intestine [11]. The retention rates of anthocyanin at various stages of digestion were meticulously assessed. Initially, 20 mg of the sample was introduced into 4 mL of simulated oral digestive fluid, which contained 2 mg/mL of α-amylase, adjusted to a pH of 6.8, and incubated at 37 °C for 5 min with agitation at 100 rpm. Subsequently, for the gastric digestion phase, the resultant mixture from the oral stage was thoroughly combined with simulated gastric juice, comprising 0.32 % pepsin, NaCl, and HCl, achieving a final pH of 2.0. This digestion mixture was then incubated under continuous stirring at 37 °C and 100 rpm for a duration of 2 h. For the simulated intestinal digestion process, the pH of the samples previously digested in gastric juice was adjusted to 7.0 using 1 M NaHCO_3_. Subsequently, the simulated intestinal digestion solution, containing 1 % trypsin and a final bile salt concentration of 5 mg/mL, was introduced. The intestinal digestion phase was maintained with continuous stirring for 4 h. At hourly intervals, equal aliquots of the digest were extracted from the reaction system to assess the retention rate. Free ACN served as the blank control. The ACN content was determined using the pH differential method, as detailed in Section 2.3. The retention rate was calculated in section 2.8.

### 2.12 Statistical analysis

Each experiment data is expressed as mean ± standard deviation (SD). The data exhibited a normal distribution, as confirmed by the Shapiro-Wilk test, and statistical significance was assessed using one-way analysis of variance (ANOVA) with Duncan’s multiple tests (*P* < 0.05) using SPSS 21.0 software (SPSS Inc., Chicago, USA).

## 3 Results and discussion

### 3.1 Physical and chemical characterization of anthocyanin complexes

**Fig. 1:**
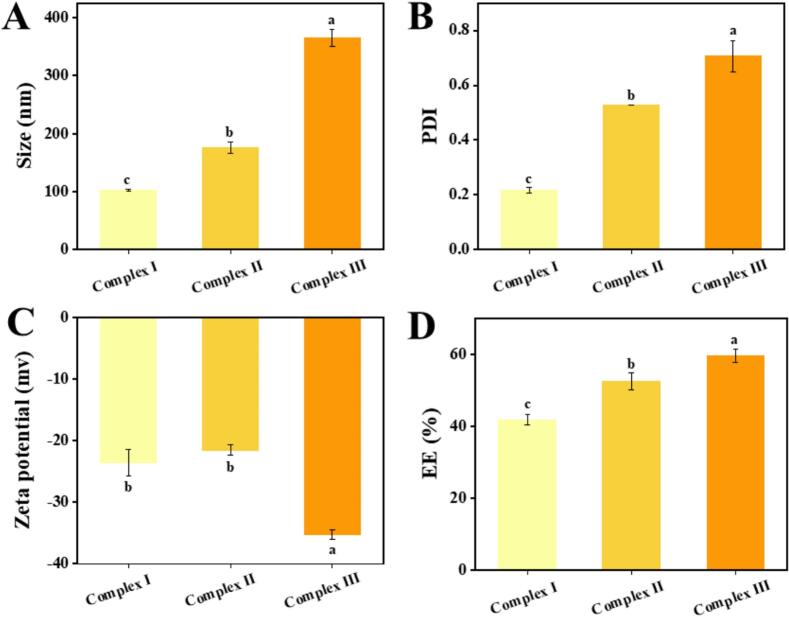
Physical and chemical characterization of anthocyanin nanocomposites. (A) Average particle size. (B) polydispersity index (PDI). (C) Zeta potential. (D) Encapsulation efficiency. Complex I, ACN@CA; Complex II, ACN@WPCA; Complex III, ACN@WPCA-FD. Different lowercase letters (a–b) represent significant intergroup differences (*P* < 0.05).

Anthocyanin complexes synthesized using CA, WPCA, and WPCA-FD are designated as Complex Ⅰ, Complex Ⅱ, and Complex Ⅲ, respectively. Fig. 1 presents the mean particle size, polydispersity index (PDI), and zeta potential values for these complexes. The particle sizes of the complexes exhibit significant differences (*P* < 0.05) (Fig. 1A), with Complex Ⅰ, Complex Ⅱ, and Complex Ⅲ measuring 102.1 nm, 175.5 nm, and 365.3 nm, respectively. In alignment with the particle size trend, Complex Ⅲ demonstrates the largest PDI (0.707) and a broader particle size distribution (Fig. 1B). This is primarily attributed to the interaction between anionic polysaccharides and proteins following the incorporation of FD into the WPCA complex, which results in an increased particle size. It is well-established that a higher zeta potential in complexes contributes to the formation of more stable systems. [25]. As illustrated in Fig. 1C, the zeta potentials of Complex I, Complex II, and Complex III are measured at –23.63 mV, −21.58 mV, and −35.25 mV, respectively. The findings indicate that the charge level of FD significantly influences the potential of the complexes. FD, being a sulfated polysaccharide, possesses a high charge density, which may facilitate its interaction with proteins to form relatively large aggregates [26]. Consequently, Complex III exhibits a high surface charge. Importantly, no statistically significant difference in potential values was observed between Complex I and Complex II (*P* > 0.05). Furthermore, the higher negative surface charge of Complex III enhances its adhesion to the inflammatory site of the colonic mucosa, thereby enabling targeted delivery of anthocyanin to the colonic region. This targeting is primarily attributed to the presence of positively charged proteins in the colonic mucosa, such as transferrin and eosinophil cationic protein [27].

Fig. 1D illustrates the encapsulation efficiency of anthocyanin across various complexes. The encapsulation efficiencies for Complex Ⅰ, Complex Ⅱ, and Complex Ⅲ were determined to be 41.71 %, 52.42 %, and 59.47 %, respectively. Notably, the binding capacity of the WPCA complex with ACN was superior to that of CA alone. This enhancement is primarily attributed to the protein–protein interactions within the WPCA Complex, where the fibrous structure is adsorbed onto the surface of the casein aggregate. The functional groups exposed on the fiber surface facilitate binding interactions with ACN, thereby improving encapsulation efficiency and loading capacity [28]. This finding underscores the high loading capability of the binary co-assembled milk protein developed in this study. Furthermore, the surface hydrophobicity of WPCA composites was significantly greater than that of CA, which further augmented the binding capacity with ACN. Conversely, Complex III exhibited a high encapsulation efficiency, suggesting that the covalent attachment of FD to WPCA significantly enhanced the loading capacity of ACN. This enhancement is primarily attributed to the incorporation of FD, a hydrophilic polysaccharide, which effectively mitigates the pronounced hydrophobicity of the protein particle surface. Consequently, this modification augments the embedding efficacy for the target compound and improves environmental stability [29]. Simultaneously, ACN is capable of forming complexes with polysaccharides through hydrogen bonding, electrostatic interactions, and hydrophobic interactions [2,30]. In addition, ACN can be incorporated into the network of complexes along with the anionic polysaccharide chains. Previous studies reported that under the strong acidic condition, the ACN solution was mainly in the form of yellow cation, and the sulfate radical in FD interacted with ACN by ion complexation, thus showing a higher degree of binding to ACN [2]. Studies have confirmed that the addition of pectin can significantly increase the loading rate of glycosylated ovalbumin on C3G [31].

### 3.2 Structure and interaction characterization of anthocyanin complexes

**Fig. 2:**
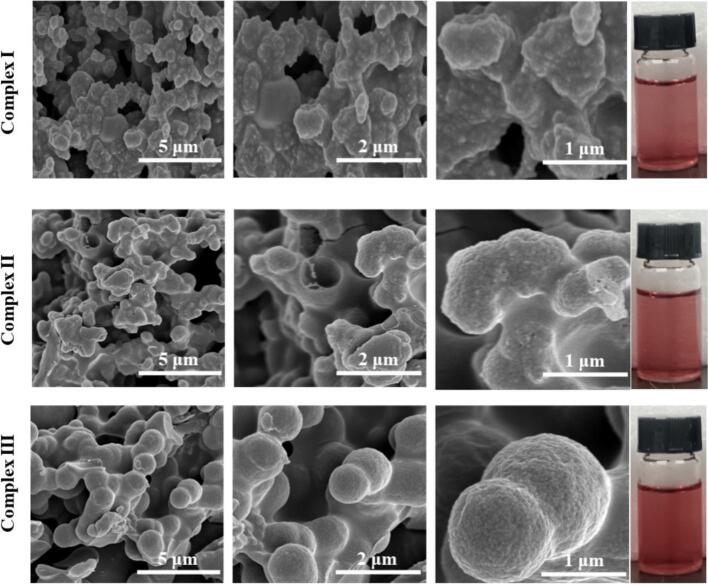
Scanning electron microscope and appearance diagram of anthocyanin nanocomplexes. Complex I, ACN@CA; Complex II, ACN@WPCA; Complex III, ACN@WPCA-FD. Scale bar indicates 5 μm for lower magnification, 2 μm for low magnification and 1 μm for high magnification.

As shown in Fig. 2, the surface morphology of anthocyanin complexes was examined using a scanning electron microscope. Notably, an adhesion phenomenon was observed between certain small and large particles in Complex I and Complex II. The surfaces of these complexes appeared relatively rough, with the agglomerates forming substantial structures characterized by large volumes and dense, irregular configurations. This phenomenon may be attributed to particle agglomeration induced by the drying process [32]. Subsequent observations revealed that, in comparison to Complex I and Complex II, Complex III exhibited a larger overall size and distinct morphology. The surface of Complex III was characterized by a uniform, regularly spherical shape with a relatively smooth texture. The capsule wall appeared to be relatively intact and compact, with consistent wall thickness. It is noteworthy that Complex I, Complex II, and Complex III all displayed a transparent and clear red liquid state, suggesting that these three nanocomplexes maintained a uniform, clear, and non-precipitating condition.

**Fig. 3:**
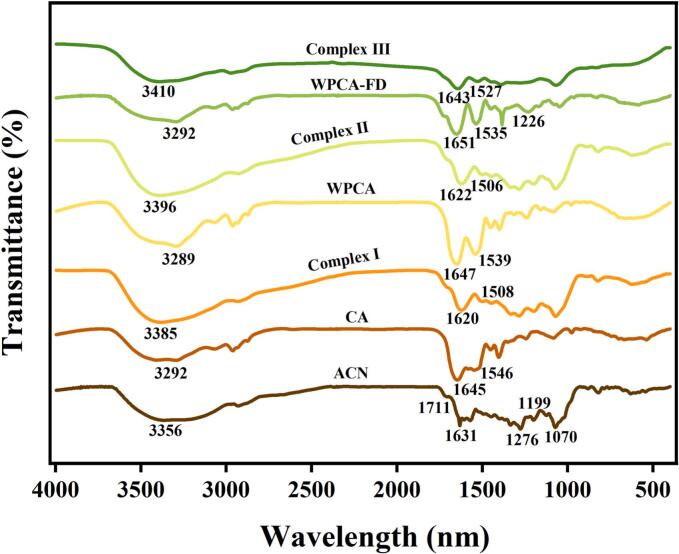
Fourier transform infrared spectra of different samples. CA, casein; whey protein isolate nanofibrils-casein complexes (WPCA); FD, fucoidan; Complex I, ACN@CA; Complex II, ACN@WPCA; Complex III, ACN@WPCA-FD.

The interactions among the three complexes during their formation were examined using Fourier Transform Infrared Spectroscopy (FTIR). As illustrated in Fig. 3, the FTIR spectrum of ACN reveals characteristic peaks at 1711 cm^−1^ and 1631 cm^−1^, which are attributed to the C=O stretching vibrations of the benzopyran aromatic ring [7]. The peak at 1070 cm^−1^ corresponds to the C-H deformation within the aromatic ring [33]. Additionally, the peak observed at 1199 cm^−1^ is associated with the tensile vibration of the pyran ring, indicative of typical flavonoid characteristics [34]. In comparison to CA, WPCA, and WPCA-FD, the spectra of Complex I, Complex II, and Complex III demonstrated significant red shifts in the characteristic peaks of the –OH group, with shifts occurring in the ranges of 3293–3385 cm^−1^, 3289–3396 cm^−1^, and 3292–3410 cm^−1^, respectively. Additionally, the absorption peaks exhibited notable broadening. This phenomenon can be attributed to the tensile vibration of the O-H bond resulting from intramolecular hydrogen bond cleavage, suggesting the presence of hydrogen bond interactions between the anthocyanin and CA, WPCA, and WPCA-FD. Notably, in the spectrum of complex III, the red shift of the O-H peak is particularly pronounced, accompanied by a broader characteristic peak, indicating a strong hydrogen bond interaction between ACN and WPCA-FD. This observation reveals that the hydroxyl group (–OH) in FD forms hydrogen bonds with the amino group (–NH_2_) in WPCA. This corroborates the hypothesis that the increase in hydroxyl groups following the covalent binding of WPCA to FD contributes to the telescopic vibration of C-OH, thereby accounting for the observed increase in both the width and intensity of the absorption peak [35].

Furthermore, in comparison to CA and WPCA, the amide I and amide II bands in the Complex I and Complex II chromatograms containing anthocyanin exhibited a blue shift, accompanied by a significant reduction in band intensities. This observation suggests the presence of hydrogen bonds and hydrophobic interactions between ACN and proteins, resulting in conformational alterations of the proteins. Previous research has demonstrated that whey protein, casein, and β-lactoglobulin (β-Lg) can interact with ACN through hydrogen bonding and other noncovalent interactions, thereby enhancing the stability of anthocyanin [6]. A comparable phenomenon in the FTIR spectrum was observed for the binding of cyanidin-3-glucoside (C3G) to preheated β-Lg and β-casein (β-CN) [36]. Most importantly, the intensities of the amide I and amide II bands in the chromatogram of Complex II were greater than those observed in Complex I. This suggests that the hydrophobic sites of protein fibers on the surface of the WPCA Complex interacted with the aromatic groups in ACN through hydrophobic interactions. This finding provides further evidence that the encapsulation efficiency of ACN in Complex II was superior to that in Complex I. Similarly, in the spectrum of Complex III, the amide I and amide II bands exhibited a noticeable blue shift and a reduction in band intensity. Notably, in comparison to WPCA-FD, the characteristic peak of the O=S=O stretching vibration of FD in Complex III was absent at 1226 cm^−1^. This absence may be attributed to the ionic complexation of the sulfate radical in FD with the yellow cation in ACN, aligning with findings reported in previous studies [37]. Furthermore, the majority of the characteristic peaks of ACN (1711 cm^−1^, 1631 cm^−1^, 1276 cm^−1^, 1199 cm^−1^, and 1070 cm^−1^) exhibited a reduction in intensity or were no longer detectable following their interaction with the three complexes [38]. This observation further corroborates the encapsulation of ACN within the three complexes.

### 3.3 Thermal stability and antioxidant activity of anthocyanin complexes

**Fig. 4:**
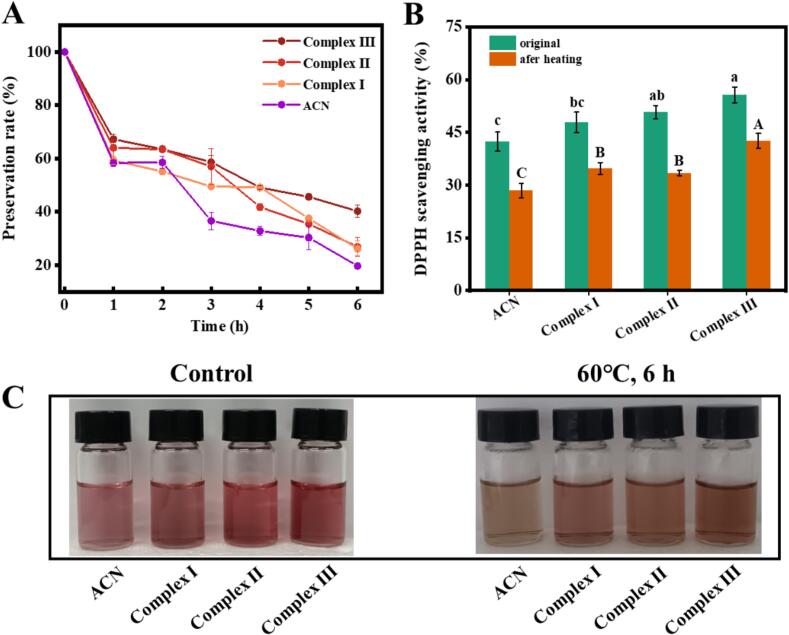
Thermal stability changes of different samples. (A) Preservation rate of ACN. (B) DPPH clearance before and after 6 h treatment. (C) appearance chart. Complex I, ACN@CA; Complex II, ACN@WPCA; Complex III, ACN@WPCA-FD. Different lowercase (a–c) or uppercase (A–C) letters represent the significant differences (*P* < 0.05).

Heat treatment is a widely employed pretreatment technique in food processing [7]. However, free ACN exhibited poor thermal stability, and exposure to elevated temperatures can lead to significant degradation. The thermal stability of ACN, along with Complex I, Complex II, and Complex III, is illustrated in Fig. 4A. Over time, the degradation of unencapsulated ACN becomes pronounced, with a retention rate of merely 19.71 % after 6 h. It is generally accepted that increased temperatures can result in the cleavage of the ACN glycosidic bond, thereby altering its structure and accelerating its degradation [39]. In comparison to unencapsulated ACN, the retention rates of Complex I, Complex II, and Complex III after 6 h of heating were 26.15 %, 26.89 %, and 40.24 %, respectively. These rates were significantly higher than those observed for free ACN, suggesting that the encapsulation effect of the three wall materials substantially enhanced the thermal stability of ACN. This improvement is primarily attributed to the noncovalent interactions, such as hydrogen bonds, formed between anthocyanin and the wall materials. These interactions necessitate the absorption of additional energy to disrupt the noncovalent bonds, thereby enhancing the thermal stability of anthocyanin [12].

The retention rate of Complex III was notably high, suggesting that glycosylated proteins can effectively mitigate the degradation of ACN. This phenomenon is closely associated with the strong anthocyanin-binding capacity of the complex, primarily attributed to the electrostatic repulsion conferred by the polysaccharides on the protein surface and the steric effects that hinder complex aggregation and enhance thermal stability. These findings align with previously reported literature [40]. In addition, after 6 h of heat treatment, the DPPH scavenging efficiency of Complex III was significantly higher than that of ACN and the other two complexes (*P* < 0.05) (Fig. 4B). The reason for this phenomenon might be that the protection of glycosylated proteins could reduce the probability of ACN contact reaction with the outside world and delay its oxidative decomposition [16]. As illustrated in Fig. 4C, the ACN solution exhibited a noticeably darker hue with a slight yellow tint following thermal exposure. Conversely, three complexes demonstrated a protective effect on the color of ACN. These findings suggest that encapsulating ACN with various wall materials mitigates oxidative degradation resulting from environmental exposure, thereby enhancing its thermal stability and antioxidant properties.

### 3.4 Storage stability of anthocyanin complexes

**Fig. 5:**
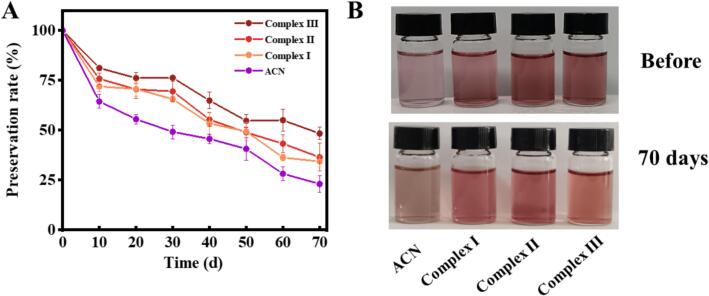
Storage stability changes of different samples. (A) Preservation rate of ACN. (B) Appearance chart. Complex I, ACN@CA; Complex II, ACN@WPCA; Complex III, ACN@WPCA-FD.

The alteration in the storage stability of ACN from various complexes was assessed by monitoring changes in the ACN retention rate during storage at ambient temperature. As illustrated in Fig. 5A, after 70 days of storage, the retention rate of ACN was 23.05 %. In contrast, the retention rates of ACN in the three complexes were significantly higher than that of the free ACN solution, suggesting that the incorporation of wall materials can mitigate the degradation of anthocyanin and reduce nutrient loss. This finding is corroborated by the visual changes depicted in Fig. 5B. Importantly, three complexes enhanced the storage stability of ACN, albeit with varying degrees of effectiveness. Complex II exhibited slight precipitation and stratification post-storage. In contrast, Complex III, which is based on glycosylated protein, demonstrated superior protective capabilities, likely due to its high encapsulation efficiency [10,12]. This suggests that ACN interacts with glycosylated protein through oxygen bonds, ionic bonds, and similar interactions, which inhibit the ring-opening cleavage of ACN [37]. Consequently, more energy is required to disrupt these non-covalent bonds, thereby enhancing the storage stability of ACN.

### 3.5 *In vitro* release of anthocyanin complexes in the simulated gastrointestinal tract

**Fig. 6:**
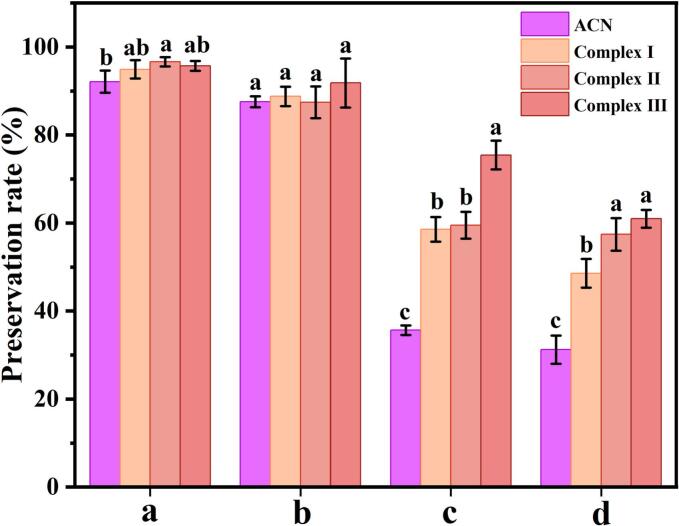
Stability of ACN during simulated digestion of different samples. (a) simulated salivary fluid, (b) simulated gastric fluid, (c) simulated intestinal fluid 2 h, and (d) simulated intestinal fluid 4 h. Complex I, ACN@CA; Complex II, ACN@WPCA; Complex III, ACN@WPCA-FD. Different letters indicate significant difference between different treatment groups of the same substance (*P* < 0.05).

Anthocyanin demonstrated stability under acidic conditions but underwent rapid degradation in near-neutral or basic environments, with the degradation rate escalating as pH levels increased [41]. The *in vitro* digestion stability of three nanocomplexes was assessed by comparing the retention rate of ACN. As illustrated in Fig. 6, following the simulated gastric digestion phase (0–2 h), there was no significant difference in ACN retention rates among the groups (*P* > 0.05). This lack of significant variation is attributed to ACN predominantly existing as a yellow cation at low pH, which enhances its stability [8]. During the simulated intestinal digestion period (2–6 h), ACN retention was markedly diminished across all samples. Notably, aqueous ACN underwent rapid degradation after 4 h of digestion in intestinal fluid, resulting in a final retention of merely 31.23 %.

Previous studies have attributed this susceptibility of ACN to oxidation and decomposition during the intestinal digestion phase to the neutral pH environment and the presence of trypsin and bile salts [42]. In comparison to the ACN solution, the retention rates of ACN in Complex I, Complex II, and Complex III were 48.57 %, 57.42 %, and 60.96 %, respectively. This suggests that ACN encapsulated within the nanocomplexes is released more gradually during gastrointestinal digestion, thereby enhancing its digestive stability. Notably, Complex III exhibited superior retention and digestive stability, rendering it particularly suitable for ACN encapsulation and transport. This enhanced performance can be attributed to three factors. The primary reason is that the ACN interacts with the protein carrier within the system through hydrogen bonding and hydrophobic interactions, which provides better protection against external environmental factors, thereby achieving high encapsulation efficiency of anthocyanin. A secondary factor is that glycosylation products can enhance the stability of the protein delivery system by augmenting spatial resistance and restricting the penetration of digestive enzymes into the protein [43]. A final consideration is that the sulfate radical in FD interacts with the yellowing cation in ACN through ion complexation, thereby preventing the conversion of ACN into an unstable structure and further achieving the objective of delaying the degradation of anthocyanin [37].

## 4 Conclusion

In conclusion, this study successfully developed anthocyanin (ACN)-loaded complexes using casein (CA), dual milk-derived proteins (WPCA), and a cold plasma-modified WPCA-fucoidan (FD) conjugate. Among the synthesized complexes, Complex III (ACN@WPCA-FD) demonstrated superior performance, exhibiting high encapsulation efficiency, enhanced thermal and storage stability, and improved digestive stability. The formation of these complexes was driven by hydrogen bonding and hydrophobic interactions, with a particularly strong hydrogen bond interaction observed between ACN and the WPCA-FD conjugate. These findings imply a potential for enhanced stability and suggest structural characteristics that may facilitate improved bioavailability; however, direct absorption studies are necessary to substantiate these claims. However, further research is required to compare the advantages and limitations of ultrasonic treatment with those of traditional encapsulation methods. Furthermore, the absence of retention experiments involving mice represents a limitation in the application of ACN complexes of glycated dual milk-derived proteins and fucoidan for the treatment of ulcerative colitis, should be a focus of future research, conducting animal experiments could provide additional evidence to support the validation of digestive characteristics. This study provides a promising strategy for the application of anthocyanins in functional foods and nutraceuticals, paving the way for future research on advanced encapsulation technologies. Nonetheless, future research should concentrate on assessing the bioavailability of encapsulated anthocyanins by implementing this technique in commercial food products.

## CRediT authorship contribution statement

**Shuangshuang Wang:** Writing – original draft, Visualization, Software, Methodology, Investigation, Data curation. **Zhenzhu Li:** Resources, Methodology. **Qingshuang Qiu:** Methodology, Data curation. **Huilin Lv:** Resources, Formal analysis. **Haokun Zhang:** Investigation, Methodology. **Hang Wang:** Methodology. **Rongzu Nie:** Resources, Data curation. **Wupeng Ge:** Writing – review & editing, Visualization. **Peifeng Li:** Supervision, Project administration.

## Declaration of competing interest

The authors declare that they have no known competing financial interests or personal relationships that could have appeared to influence the work reported in this paper.
